# Making the Case for Open Dental Education

**DOI:** 10.1002/jdd.70132

**Published:** 2026-01-16

**Authors:** Casey D. Wright, Pamela P. Smalley, Fanny Eliza Bondah, Jennifer L. Lehto, Tonia Morley, Danelle Orange, Elisabeta Karl, Royce Kimmons

**Affiliations:** ^1^ School of Dental Medicine Pacific Northwest University of Health Sciences Yakima Washington USA; ^2^ Department of Instructional Psychology and Technology Brigham Young University Provo Utah USA; ^3^ Chippewa Valley Technical College Eau Claire Wisconsin USA; ^4^ Milwaukee Area Technical College Milwaukee Wisconsin USA; ^5^ Raynor Library Marquette University Milwaukee Wisconsin USA; ^6^ School of Dentistry Marquette University Milwaukee Wisconsin USA; ^7^ Department of Educational Leadership and Foundations Brigham Young University Provo Utah USA

## Abstract

Dental education as a field and dentistry as a profession are facing significant challenges, including workforce shortages, ever‐rising costs, unmet student and community needs, as well as quality of pedagogical practices. Open educational resources are learning objects that are freely accessible and are able to be retained, reused, revised, remixed, and redistributed under the philosophy of more openness and sharing. While some efforts are being made to address the aforementioned concerns, the prospects of more readily welcoming and adopting open education have not been discussed as a potential means of alleviating these barriers. Here, we expound on some of the challenges facing dental education and how open educational resources can help address these challenges. Given the reduced barriers and technological advantages, open educational resources and the adoption of more openness in dental education can alleviate some of the individual, institutional, and overall challenges faced by the field of dentistry and the oral healthcare enterprise as a whole.

## 1 Introduction

Dental education is at an important crossroads, with significant challenges impacting the equitable accessibility and quality of training for future oral health providers, including dentists, dental hygienists, dental therapists, dental assistants, and expanded function dental auxiliaries. Contributing factors to these challenges include the escalating costs of dental education (e.g., tuition, textbooks, other materials like board examination study aids, and loupes/equipment), and the cost of living, along with the use of outdated pedagogical practices or faculty without training in contemporary educational practices, and misalignment with student and community needs. Issues of accessibility of content and opportunities only accentuate provider shortages in rural and other underserved areas, in part, by making opportunities for individuals from these areas unattainable. Not addressing these challenges could lead to widening gaps in oral healthcare services and exacerbate inequitable oral health outcomes. In this article, we will discuss the context of these concerns and argue that embracing a philosophy of openness within dental education, particularly the promotion of open educational resources (OER), can play a valuable role in overcoming these challenges. We first outline several of the pressing problems and challenges facing dental education. We then describe the nature of OER and discuss how it can help to address the outlined problems and challenges.

## 2 Problems and Challenges

### 2.1 Problem of Demand and Workforce Shortages

The dental profession is experiencing a workforce problem, with a notable shortage of both dental providers and dental educators. There are ongoing conversations about the potential shortage of dentists, particularly in rural and underserved communities (i.e., DDS/DMD degree holders). Workforce shortages have also been noted among dental hygienists, which has increased since the COVID‐19 pandemic [1]. These shortages affect the ability to meet the growing demand for dental services, especially in communities that need them the most. In terms of dental educator shortages, there has been a 78% increase in full‐time dental faculty shortages and a 118% increase in part‐time faculty shortages since the year 2000 [2]. Resultant student‐faculty ratios, low clinic coverage, and limited availability of instructors negatively impact the quality and experience for aspiring providers and trainees.

### 2.2 Problem of Cost for Dental Education and Materials

Shortages in the number of available dental educators are related, in part, to dentists early in their career who might be interested in an academic career grappling with student loans and the high costs of their own dental education, and the lower earning potential of dental faculty relative to working in private practice [3]. Indeed, the educational costs of becoming a dental provider continue to rise, and it feels like little is being done to address this problem. Istrate et al. estimate the average debt for obtaining a DDS or DMD degree in the United States at $286,200. Of note, around 20% of those in a predoctoral dental program report having support from family to pay for dental school, potentially creating a different overall experience for those with such support (e.g., less worry about housing, food, or other basic needs) [4]. The cost of dental education has also risen more quickly than the earning potential of dentists. The American Dental Education Association (ADEA) reports the average total program cost for dental hygiene programs to be between $22,692 and $36,382 (2023). In addition, dental hygiene programs are typically 2‐year programs offered by community or technical colleges, which historically include higher enrollment of students from underserved and minoritized populations [5].

Cost burdens on students are not limited to tuition, but also include textbooks, materials, equipment, and other resources. In addition, the overall cost of living for students also continues to rise [6]. This is particularly relevant to dental hygiene programs, where the cost of textbooks can encompass a greater proportion of the overall cost of training, meaning efforts to reduce textbook costs can create meaningful change for hygiene program affordability. Regarding student retention, learning outcomes, health, and well‐being, historically underserved students are particularly vulnerable to the extra‐tuition costs, when other unplanned or uncovered costs of dental education are considered (e.g., healthcare, healthy food, and adequate housing).

### 2.3 Problem of Disconnecting With Students and Community Needs

Dental educational content and experiences are often disconnected from the local needs of students and, ultimately, the patients to be served. Dental education curricula and materials rarely consider the unique languages, cultures, varying ability needs, and life experiences of students. When considering the accessibility of learning resources specifically, content may be presented in a language other than the learner's primary language, at a reading level or style of the highly educated person who wrote the material, in a format with poor user experience design and that is less user‐friendly, and not designed in a way to meet contemporary student lifestyle needs (e.g., on the go for those must commute, digital or mobile‐friendly). In addition, curricular experiences and materials (e.g., content, textbooks) are often targeted at a general audience and may not adequately address the unique diversity of patients and needs of communities in which dental programs find themselves (e.g., specific cultural nuances). In summary, these challenges affect students and other stakeholders in dental education and require innovative strategies for overcoming such barriers.

### 2.4 Problem of Pedagogical Quality and Learning Materials

While the direct clinical experience of many dental educators is invaluable, faculty members in dental programs rarely have a background in education and lack significant training in pedagogical practices or curricular development [7]. As a result, faculty members may struggle to meet the needs of learners, to identify high‐quality educational materials, or to design effective content and assessments. Faculty development efforts may help faculty in some of these skills, but such opportunities are typically sporadic, limited, and of little interest to overworked faculty, who find themselves grappling with problems of educational practice relatively alone.

## 3 Open Education as a Solution

OER offers some promise to the educational challenges by promoting equity, affordability, and innovation. OER are openly licensed materials that allow free use, adaptation, and sharing while also maintaining the possibility of rigor and quality (e.g., through peer review, expert authors, and continuous updates). Based on Wiley's 5 R Framework [8], OER can be retained, reused, revised, remixed, and redistributed. As a result, these resources reduce or eliminate the financial burdens of costly textbook expenses on students, particularly first‐generation and underserved learners [9]. Recognized for their quality and adaptability, OER improve student outcomes and empower educators to create content for diverse needs [10]. Though challenges remain, particularly in under‐resourced and non‐English‐speaking regions, fostering awareness and supportive attitudes is key to expanding the impact of OER [11].

Attempts to create or adopt OER within dental education have been very scarce. Similarly, to our knowledge, no known organized effort has previously existed to compile readily available open content to be used in dental education. While many scientific articles or research‐related books/chapters have been published open access, little has been done in the realm of creating or distributing content appropriate for training programs in the form of open textbooks or other materials. OER are not confined to textbooks, but could also include openly licensed and accessible instructional videos, cases, assessments, clinical procedure guides, or others.

## 4 Benefits of OER for Dental and Oral Health Education

### 4.1 Alleviating Workforce Demand and Shortages

OER can help meet the increasing demand for both dental practitioners and dental educators, particularly in underserved communities, by providing more accessibility of materials, reducing costs, and more. In many regions, access to dental education is restricted due to a lack of dental training programs and/or faculty [12]. By supporting online and hybrid learning models, OER facilitates possibilities for students to access high‐quality education without the need for additional faculty due to the scalable nature and flexibility in terms of the needed location of both faculty and students [9]. This is especially valuable in rural areas where dental programs, facilities, and educators are limited or unavailable. For example, open‐access pre‐dental courses using OER and hybrid or online formats could let students complete foundational coursework remotely while reducing the need for faculty time through scalable technology. This could be at a reduced cost in an undergraduate program or outside of the traditional pre‐dental program altogether. Because of its less restrictive licensing, OER's flexible learning pathways can support diverse students by allowing for the development of personalized platforms and the reviewing of materials as needed [9]. From this, students can gain a strong academic foundation before advancing to hands‐on clinical training.

### 4.2 Addressing the Cost of Dental Education

As noted earlier, the financial burden of dental education can deter prospective students and create additional stress for current ones. By integrating OER into a curriculum (e.g., through open textbook adoption, the inclusion of open courses), institutions can reduce the overall cost of dental programs. While OER may not directly reduce tuition costs, they can significantly alleviate the other financial burdens on dental students by eliminating or drastically reducing the cost of textbooks and other educational materials, saving institutional costs for textbook databases, and without violating copyright laws by downloading or sharing unauthorized PDFs [6]. In time, OER, in combination with other technologies, could facilitate more efficient and shorter training periods by allowing content that needs to be strictly didactic to be administered online and more freely. Shorter training periods or more efficient training could potentially lead to lower tuition costs for students as well.

OER also enhances students' long‐term financial stability. Due to financial constraints, those from underserved communities may be discouraged from pursuing careers in dentistry or dental hygiene [5]. Lowering financial barriers by using OER could expand opportunities for a more diverse and inclusive workforce of aspiring dental professionals and improve access to dental care in communities that need it the most [13]. Reducing students' financial strain, particularly as it relates to the cost of health and well‐being, can help institutions improve retention and boost degree completion [9], and potentially assist in reducing the need for remedial experiences, though more research is needed in this area.

### 4.3 Addressing Community and Student Needs

OER can be adapted to diverse populations that students will serve, making dental education more culturally inclusive [5]. Traditional textbooks may not fully represent the unique oral health needs and challenges faced by different demographic groups, allowing educators to tailor content to the specific communities' students will be serving, which is typically not as easy or possible with traditionally copyrighted material [5]. In addition, OER helps students develop cultural competence as they are exposed to diverse scenarios, language, and vocabulary considerations, and treatments [5]. With the benefits of cultural inclusivity through OER, future dental professionals are then better prepared to meet the needs of their communities.

Specific to student needs, OER offers the ability to customize educational materials to better meet the needs of diverse learners. This flexibility allows educators to modify and adapt resources to fit specific curricular needs, cultural contexts, and student preferences without compromising the intellectual property of the original creators of the resources. For example, a teacher can update a textbook with the latest research, translate materials into different languages (either themselves or through artificial intelligence), use tools to improve the readability levels, or incorporate local case studies. This level of customization, often facilitated easily with the improvement of various technologies, ensures that educational content remains relevant, engaging, accessible to all abilities (e.g., easily creating audio versions, toggling between languages and readability levels), and ultimately enhances the learning experience for students.

### 4.4 Addressing the Need for Quality Materials to Support Effective Pedagogy

OER can also provide diverse perspectives. As dental educators redistribute, reuse, or remix open‐accessed resources, dependent on the Creative Commons licenses, students benefit by being exposed to differing viewpoints. Unlike traditional textbooks, which can quickly become outdated and may take years to be revised and republished, OER can be updated in real time [11], allowing dental educators and students to continuously update course objectives and stay current with advancements and regulatory changes [11]. Faculty with little training in education or instructional practices can also rely on materials that were created by others and easily implement them into their courses or pedagogical approaches.

By using OER, dental educators can integrate real‐world applications and emerging best practices. Educators can engage students more effectively across institutions and collaborate on new materials, incorporating feedback to improve clarity, accuracy, and effectiveness [14]. In addition, several OER platforms are moving towards providing means for content to be peer‐reviewed, ensuring the creation of quality dental educational materials [11].

## 5 Conclusion

In conclusion, there are many compelling reasons to adopt OER in dental education, from reducing student costs and improving access to enhancing curriculum flexibility and instructional quality. OER offers scalable, updatable, and collaborative solutions that address long‐standing challenges in dental programs, such as faculty shortages, by extending the scale of current faculty members' reach and outdated materials. Ongoing initiatives like ours play a critical role in promoting high‐quality, accessible OER that meet the evolving needs of dental educators and learners. As the field continues to face both financial and instructional pressures, embracing OER is not just a cost‐saving strategy but a meaningful step toward more equitable, innovative, and sustainable dental education.
